# Profiling of Volatile Compounds in ‘Muscat Hamburg’ Contaminated with *Aspergillus carbonarius* before OTA Biosynthesis Based on HS-SPME-GC-MS and DLLME-GC-MS

**DOI:** 10.3390/molecules29030567

**Published:** 2024-01-23

**Authors:** Yayun Guo, Zhe Wang, Yi He, Huanhuan Gao, Hongmei Shi

**Affiliations:** 1 Shandong Academy of Grape, Shandong Academy of Agricultural Sciences, Jinan 250100, China; guoyayun405@163.com (Y.G.); limingyingxiong@163.com (Z.W.); heyicau221@163.com (Y.H.); 2 Institute of Plant Protection, Shandong Academy of Agricultural Sciences, Jinan 250100, China; gaohuanhuan368@126.com

**Keywords:** volatile compounds, Ochratoxin A, QuEChERS-HPLC-FLD, HS-SPME-GC-MS, DLLME-GC-MS, OPLS-DA

## Abstract

*Aspergillus carbonarius* is known to produce the carcinogenic ochratoxin A (OTA) in grapes. The metabolism process before OTA biosynthesis influences the content and composition of the volatile compounds in grapes. In this study, a self-established method based on QuEChERS coupled with high-performance liquid chromatography–fluorescence detection (HPLC-FLD) was used to determine the OTA levels during a seven-day contamination period. The results showed that OTA was detected on the second day after contamination with *A. carbonarius*. Thus, the first day was considered as the critical sampling timepoint for analyzing the volatiles in grapes before OTA biosynthesis. Additionally, the volatile compounds in grapes were analyzed using headspace solid-phase microextraction gas chromatography–mass spectrometry (HS-SPME-GC-MS) and dispersive liquid–liquid microextraction gas chromatography–mass spectrometry (DLLME-GC-MS). The corresponding data were evaluated via multivariate data analysis using projection methods, including PCA and OPLS-DA. The results indicated significant differences in the nine volatile compounds in grapes contaminated with *A. carbonarius* before OTA biosynthesis. The results of the Pearson correlation analysis showed positive correlations between ethyl acetate, styrene, 1-hexanol and OTA; (*E*)-2-hexenal and nerolic acid were negatively correlated with OTA. Overall, these findings provide a theoretical basis for the early prediction of OTA formation in grape and grape products using GC-MS technology.

## 1. Introduction

Ochratoxin A (OTA), a mycotoxin produced by certain fungal species, especially Aspergillus and Penicillium, is the most toxic member of the ochratoxin family. It is widely found in grapes and their derived products, including grape juice, raisins, and wine [1,2,3]. OTA has been shown to exert potential teratogenic, mutagenic, carcinogenic, and immunosuppressive effects in humans and animals [4]. It has also been classified as a possible human carcinogen (group 2B) by the International Agency for Research on Cancer [4]. Although OTA is less toxic than aflatoxin, it serves as a valuable model toxin for scientific research. *Aspergillus carbonarius* is recognized as the major OTA-producing fungal species in grapes and their derived products [5,6,7,8]. The contamination of grapes with *A. carbonarius* leads to the production of secondary metabolites such as volatile compounds during or before OTA production. Notably, the synthesis of volatile compounds is associated with mycotoxin production [9,10]. Thus, detecting the changes in contents and types of volatile compounds in grapes contaminated with *A. carbonarius* is of great significance for early OTA detection, to ensure the quality and safety of grapes and their derived products.

Currently, high-performance liquid chromatography coupled with fluorescence detection (HPLC-FLD) is widely adopted for detecting the content of OTA due to its high sensitivity and reproducibility [11]. However, it is imperative to perform sample extraction before HPLC-FLD analysis due to the complexity of the fresh or contaminated grape matrix. Among the sample extraction and preparation techniques, the QuEChERS (Quick, Easy, Cheap, Effective, Rugged, Safe) method has gained increasing attention regarding the determination of mycotoxin residues in various matrices because it is easy to operate, rugged, and safe; it also has a reduced number of procedural steps and can effectively eliminate interfering compounds from samples [12,13,14]. Additionally, the QuEChERS method has the advantages of rapidity, cost-effectiveness, and high efficacy [15]. Thus, the QuEChERS-HPLC-FLD method was used for the detection of OTA in this study.

The headspace solid-phase microextraction (HS-SPME) technique is a solvent-free extraction method that combines extraction and preconcentration into a single step [16]. Furthermore, GC-MS is the main technology used to determine flavor or volatile profiles. Recently, HS-SPME coupled with gas chromatography–mass spectrometry (HS-SPME-GC-MS) has gained significant popularity for analyzing volatile compounds. HS-SPME-GC-MS is generally used for the determination of very volatile compounds. Moreover, it has been used to determine the off-flavor in wine [17], microbial volatile organic compounds [18], the volatile composition of grapes [19,20], pesticide residues, and veterinary drug residues in foods [21,22].

However, the HS-SPME mode cannot effectively extract fewer volatile and highly water-soluble compounds for GC analysis [23]. Dispersive liquid–liquid microextraction (DLLME), a relatively new sample preparation method developed by Rezaee et al. [24], can overcome the aforementioned limitation. DLLME has been widely employed for analyzing various chemical compounds in different types of matrices due to its advantages of simplicity, rapidity, cost-effectiveness, and environmental benignity [25]. For instance, DLLME coupled with GC-MS has been used for analyzing volatile compounds in food matrices [23,26]. Thus, DLLME-GC-MS can complement HS-SPME-GC-MS for aroma profiling, providing a more comprehensive analysis of the volatile compounds in grapes.

The present study aimed to achieve the following: (i) Develop a QuEChERS-HPLC-FLD method for OTA determination in grapes contaminated with *A. carbonarius* using low-cost graphite powder as the adsorbent. In particular, the performance of the extraction solvents and cleaning sorbents and the experimental factors influencing the extraction efficiency were evaluated, followed by a comprehensive comparison of the traditional adsorbents with graphite powder. (ii) Analyze the volatile compounds in fresh/contaminated grapes using HS-SPME-GC-MS and DLLME-GC-MS. (iii) Explore the changes in volatile compounds in grapes contaminated with *A. carbonarius* before OTA biosynthesis to mimic the real-life scenarios. The experimental findings can provide valuable insights for establishing a potential method for the early detection and monitoring of OTA contamination and for identifying the volatile markers of fungi/OTA in grapes.

## 2. Results and Discussion

### 2.1. OTA Detection in Grapes

**Figure 1:**
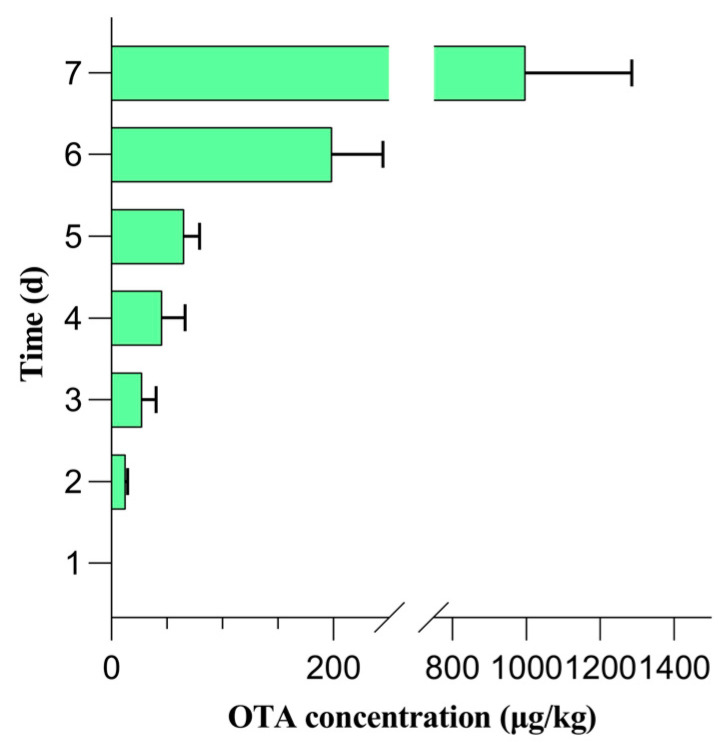
Changes in ochratoxin A (OTA) contents during a seven-day contamination period in QuEChERS-HPLC.

The QuEChERS-HPLC method was applied to analyze the OTA-free fresh grapes, grapes in the CK group, and contaminated grapes. The changes in the OTA levels during a seven-day contamination window are shown in Figure 1. OTA was not detected in the contaminated grapes on days 0 and 1, but was detected on day 2, indicating that OTA can be detected in grapes within two days of contamination with *A. carbonarius*. The OTA content gradually increased from day 2 to day 7 in the contaminated sample group. However, OTA was not detected in the CK sample group throughout the seven-day contamination window. Hence, the optimized method was used for OTA detection in fresh grapes. Notably, OTA was not detected in any of the fresh grape samples. Thus, day 1 was considered as the critical sampling timepoint for analyzing the volatile compounds in grapes before OTA biosynthesis.

### 2.2. Volatile Compound Profiling in Grapes Prior to OTA Biosynthesis

#### 2.2.1. PCA of Volatile Compounds

In this study, 56 volatile compounds were identified by HS-SPME-GC-MS, including alcohols, carbonyl compounds, esters, acids, terpenes, olefins, and others (Supplementary Table S1). Additionally, 15 volatile compounds were identified by DLLME-GC-MS (Supplemental Table S2). Generally, the types of compounds changed after cultivation with *A. carbonarius*, but only the contents of volatile compounds changed, as shown in Tables S1 and S2. It is possible that, as this was the early stage of infection, the volatile compounds were less altered and there was no change in their types.

**Figure 2:**
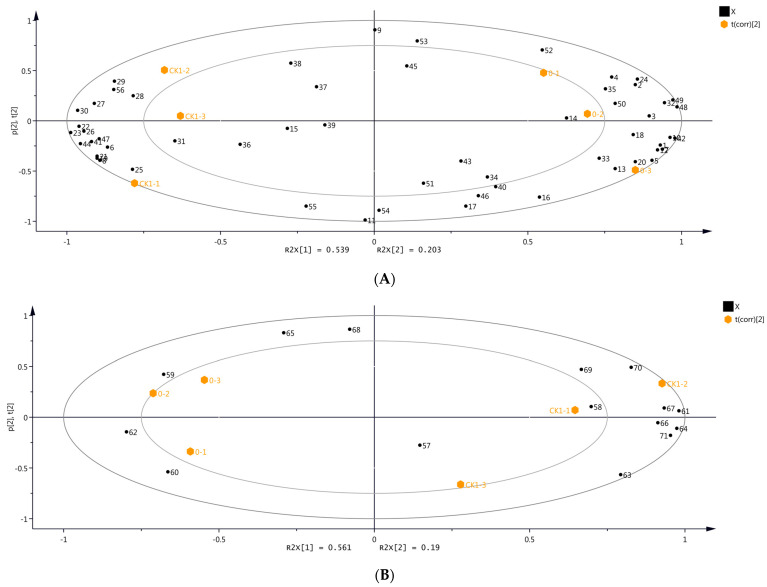
Principal component analysis (PCA) with volatile compounds identified in grape samples from day 0 and CK1 using two identification methods. (**A**) HS-SPME-GC-MS. (**B**) DLLME-GC-MS. Numbers refer to the volatile compounds, as listed in Tables S1 and S2 (Please refer to Figure S1 for the overlapping content).

The differences in the volatile compounds among the grape samples were evaluated using unsupervised PCA. The first principal component (PC1) and the second principal component (PC2) contributed to 53.9% and 20.3% variations in the samples, respectively, representing 74.2% of all variance (Figure 2A). PC1 and PC2 provided a large amount of information on the samples from day 0 and CK1, which can reflect the overall volatiles profile of grapes. The samples from day 0 and CK1 did not cluster together (Figure 2A), indicating significant differences in the volatile compounds between these samples. Subsequently, PCA was performed on the volatile compounds of the samples from day 0 and CK1 identified by DLLME-GC-MS (Figure 2B). PC1 and PC2 contributed to 56.1% and 19.0% variations in the samples, respectively, providing comprehensive and substantial information about these samples. The volatile compounds of the samples from day 0 and CK1 differed significantly (Figure 2B). Therefore, the CK1 sample from day 1 was selected as the control for analyzing the volatile compounds in grapes before OTA biosynthesis.

**Figure 3:**
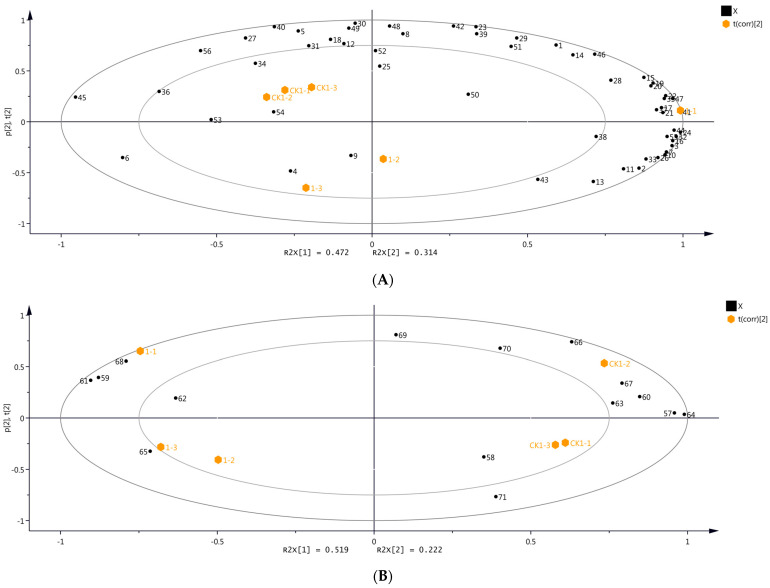
Principal component analysis (PCA) with volatile compounds identified in grape samples from day 1 and CK1 using two different identification methods. (**A**) HS-SPME-GC-MS. (**B**) DLLME-GC-MS. Numbers refer to the volatile compounds, as listed in Tables S1 and S2 (Please refer to Figure S2 for the overlapping content).

Figure 3 depicts the results of the PCA based on the volatile compounds detected through HS-SPME-GC-MS and DLLME-GC-MS, indicating significant differences in the volatile compounds before OTA biosynthesis. Accordingly, the accumulated contributions of PC1 and PC2 were 78.6% and 74.1% for HS-SPME-GC-MS (PC 1: 47.2%; PC 2: 31.4%) and DLLME-GC-MS (PC 1: 51.9%; PC 2: 22.2%), respectively, providing adequate information about the samples and their characteristic volatiles. The volatile compounds in samples CK1 and day 1 differed significantly (Figure 3).

#### 2.2.2. OPLS-DA of Volatile Compounds and Correlation of OTA with Volatiles

OPLS-DA is a supervised method used for classifying samples, constructing discriminant models, and eliminating the influence of irrelevant data [27,28]. In this study, OPLS-DA was performed on the volatile compounds of samples from day 1 and CK1 to identify the significant differential volatiles in grapes contaminated with *A. carbonarius* before OTA biosynthesis.

**Figure 4:**
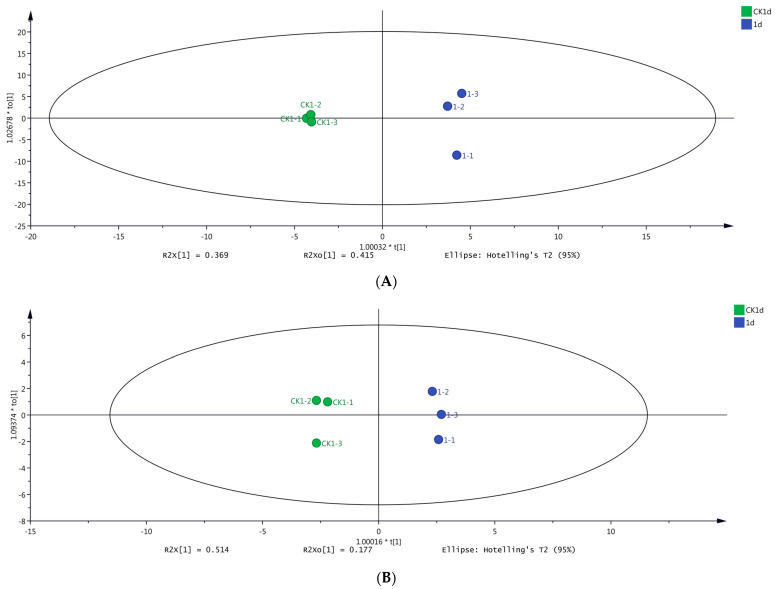
Orthogonal partial least squares discriminant analysis (OPLS-DA) score plots of grape samples on day 1 and CK1 determined using two different methods. (**A**) OPLS-DA score plot of samples on day 1 versus sample CK1 based on HS-SPME-GC-MS data. (**B**) OPLS-DA score plot of samples on day 1 versus sample CK1d based on DLLME-GC-MS data.

Based on the results of HS-SPME-GC-MS (Figure 4A) and DLLME-GC-MS (Figure 4B), there was no overlap between samples CK1 and day 1, indicating excellent discrimination among samples. The volatile compounds in samples CK1 and day 1 were clustered separately on the x-axis of the OPLS-DA model. Notably, the best classification result was achieved by the OPLS-DA model, indicating significant differences in the volatile compounds between samples CK1 and day 1.

R^2^ and Q^2^ indicate the explanation ability and the prediction ability of the OPLS-DA model, respectively [29]; R^2^ > 0.5 and Q^2^ > 0.5 are indicative of an OPLS-DA model with suitable interpretation and prediction abilities [30]. In the present study, these coefficients were R^2^X = 0.784, R^2^Y = 0.996, and Q^2^ = 0.959 (Figure 4A), and R^2^X = 0.691, R^2^Y = 0.994, and Q^2^ = 0.977 (Figure 4B), collectively indicating two stable and reliable models. 

Moreover, a significant difference in volatile compounds was observed between the samples CK1 and day 1, allowing them to be classified into two groups (at 95.0% confidence interval).

**Figure 5:**
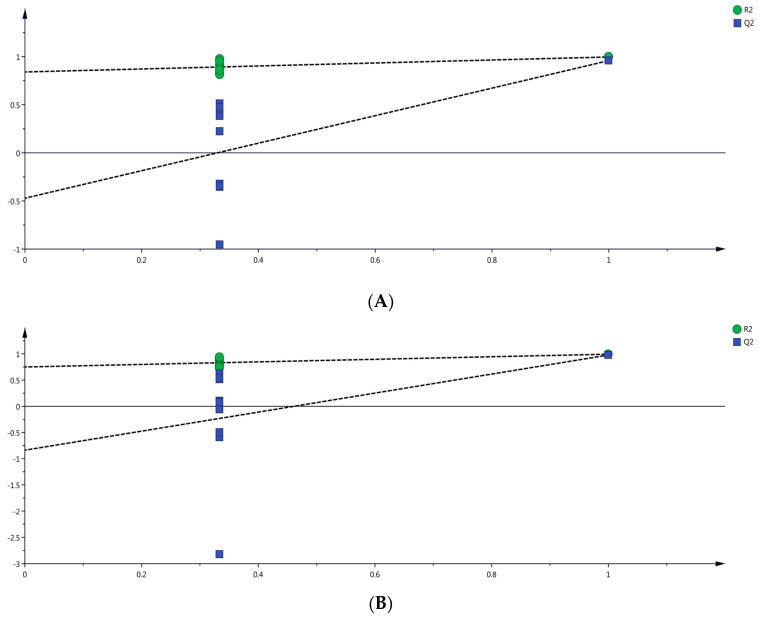
Verification diagram based on orthogonal partial least squares discriminant analysis (OPLS-DA). (**A**) by HS-SPME-GC-MS. (**B**) by DLLME-GC-MS.

Furthermore, a permutation test with 200 iterations was conducted to validate the fitting degree of the OPLS-DA model (Figure 5). The intercept values of R^2^ and Q^2^ in the permutation models were lower than the original values, and the slope of the regression line was steep. Moreover, the intercept of Q^2^ was negative, indicating that the two models did not overfit. 

The significant differential volatiles in the grape samples from day 1 and CK1 were analyzed using variable importance in projection (VIP) analysis. The VIP values indicate the variable contribution rate to the classification, with VIP values > 1 indicating a key variable in the discriminant model [31]. The significant differential volatiles were selected based on the *p*-values from *t*-tests (*p* < 0.05).

Table 1 summarizes the significant differential volatiles in samples CK1 and day 1 based on the HS-SPME-GC-MS and DLLME-GC-MS data. A total of seven and three volatile compounds were selected as significant differential volatiles in samples CK1 and day 1. In the experimental group, a significant increase was observed in trans-2-(2-pentenyl)furan, 1-hexanol, and 3,7-dimethyl-3,6-octadienal compared to CK1, while other significant differential volatiles showed a downward trend in the samples from day 1. 

Notably, (*E*)-2-hexenal, nerol and nerolic acid were detected in the samples using both methods. Other significant differential volatiles were uniquely found based on the HS-SPME-GC-MS or DLLME-GC-MS data. Nerol was also identified using both methods; this was a significant differential volatile that was only in the analysis conducted based on the DLLME-GC-MS data, possibly due to its weak polarity. Nerol was better extracted when the weakly polar dichloromethane was used as the extractant in DLLME-GC-MS analysis. Nerol was adsorbed very lightly by the 50/30 µm DVB/CAR/PDMS fiber, which was due to the incomplete adsorption of nerol. Thus, no significant difference was observed in the nerol content among the grape samples based on HS-SPME-GC-MS. 

**Figure 6:**
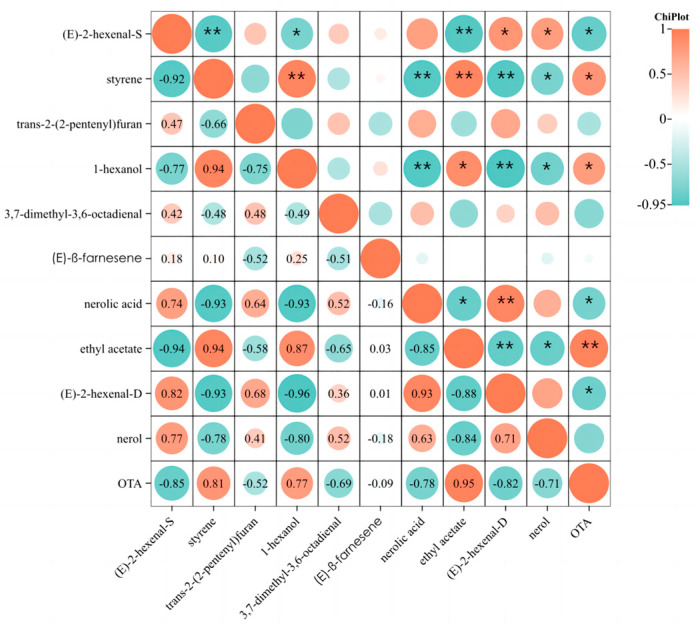
Heatmap of correlation between OTA and significant differential volatile compounds in grape samples CK1 and day 1. The suffixes “S” and “D” for (*E*)-2-hexenal, respectively, refer to (*E*)-2-hexenal detected using HS-SPME-GC-MS and DLLME-GC-MS methods. ** Correlation is significant at the 0.01 level. * Correlation is significant at the 0.05 level.

Pearson correlation analysis was employed to investigate the correlation between OTA and significant differential volatile compounds over a seven-day contamination period, as shown in Figure 6. The obtained results showed positive correlations between OTA and ethyl acetate (*p* < 0.01), styrene (*p* < 0.05) and 1-hexanol (*p* < 0.05); OTA was negatively correlated with (*E*)-2-hexenal (*p* < 0.05) and nerolic acid (*p* < 0.05). Grapes without *A. carbonarius* presented higher levels of (*E*)-2-hexenal and nerolic acid. In contrast, higher levels of volatiles, such as ethyl acetate, styrene, and 1-hexanol, were found in grapes inoculated with *A. carbonarius*. In addition, trans-2-(2-pentenyl)furan, 3,7-dimethyl-3,6-octadienal, (*E*)-β-farnesene and nerol had little correlation with the OTA secretion in *A. carbonarius*-cultured grapes. With the growth of *A. carbonarius* in the inoculated grapes, the content of ethyl acetate, styrene and 1-hexanol gradually increased; meanwhile, the content of (*E*)-2-hexenal and nerolic acid gradually decreased.

The metabolism of OTA in grapes can be affected by several factors, such as the surrounding environment, the geographical location of vineyards, the soil composition, and the grape cultivar [32]. Some scholars have researched the markers of *A. carbonarius* contamination in grapes. For example, Schueuermann [33] found that the presence of ethyl acetate was indicative of *A. carbonarius* contamination in Chardonnay grapes in Australia. In addition, 1-hexanol and (*E*)-2-hexenal were also identified as markers when Cabernet Sauvignon and Moscato Italico grapes were inoculated with *A. carbonarius* for 6 days in Brazil [34].

## 3. Materials and Methods

### 3.1. Chemicals and Reagents

Magnesium sulfate and sodium chloride were purchased from Sinopharm Chemical Reagent Co., Ltd. (Beijing, China). GC-grade dichloromethane (≥99% purity) was purchased from Merck Chemical Co. Inc. (Darmstadt, Germany). OTA and 4-methyl-2-pentanol were purchased from Sigma-Aldrich (St. Louis, MO, USA). n-Alkanes (C7–C30) were obtained from Supelco (Bellefonte, PA, USA). Primary secondary amine (PSA), C18 (octadecyl) and graphitized carbon black (GCB) were purchased from Agela Technologies (Tianjin, China). Graphite powder (99.8%) was purchased from Shanghai Aladdin Biochemical Technology Co., Ltd. (Shanghai, China). HPLC-grade acetonitrile (ACN) and formic acid (99% purity) were purchased from Merck (Darmstadt, Germany). Ultra-pure water was obtained using a Milli-Q purification system (Millipore, Bedford, MA, USA).

### 3.2. Grape Samples

The grape berries (*Vitis vinifera* L. ‘Muscat Hamburg’) (17.8 °Brix, pH = 4.11) included in this study were harvested in 2022 in the Pingdu region (latitude, 36.99 N; longitude, 119.93 E), Shandong, China. Only healthy grape berries without obvious signs of physical damage or fungal growth were included in this study.

### 3.3. A. carbonarius and Culture Conditions

The *A. carbonarius* CCTCC AF2015026 strain was procured from the China Center for Type Culture Collection (CCTCC). The fungal spores cultured on potato dextrose agar (PDA) at 28 °C for 5 days were rinsed with sterile saline solution, and the concentration was adjusted to 1 × 10^6^ spores/mL using a hemocytometer.

### 3.4. Grape Inoculation and Incubation Conditions

Grape berries were separated from bunches by cutting the stem at approximately 1 cm from each grape. The berries were sterilized according to the previously reported method [35]. Two microliters of the *A. carbonarius* conidial suspension (1 × 10^6^ conidia/mL) were inoculated into each grape berry at a depth of approximately 2 mm below the skin using a sterile microsyringe. Six inoculated berries (50 g ± 1 g) were placed initially in a Petri dish and then in a growth chamber under controlled temperature (25 °C) for seven days. Eight sampling points were considered, i.e., day 0, 1, 2, 3, 4, 5, 6, and 7, and the experiment was conducted in triplicate. Grapes that were inoculated with sterile distilled water without the addition of conidial suspension and subjected to the same experimental conditions were considered as the control (CK).

### 3.5. Optimization of the QuEChERS Procedure

The extraction and purification steps are regarded as the most crucial stages in the QuEChERS method for OTA determination, particularly for complex matrices [12]. Considering the complex matrix of grape or contaminated grapes and the presence of numerous compounds, the optimization of the extraction and purification steps is necessary to maximize the OTA extraction efficiency. Thus, OTA-free Merlot berries were selected as the model matrix. In the optimization experiment, the extraction efficiency was determined by the peak area of OTA.

#### 3.5.1. Selection of Extraction Solvent

Generally, the recoveries of analytes are influenced by the extraction solvent [36]. ACN has a superior phase separation ability during liquid–liquid partition and salting-out when used for extracting mycotoxins [12,37,38]. The presence of acid in the extraction solvent is crucial for extracting acidic toxins (including OTA), especially formic acid [12]. Therefore, the effect of different extraction solvents, i.e., ACN, ACN/formic acid (99.9/0.1, *v*/*v*), ACN/formic acid (99/1, *v*/*v*), and ACN/formic acid (95/5, *v*/*v*), on the OTA extraction efficiency was compared. The OTA peak areas were determined by adding OTA standard solutions to the blank samples. The OTA-spiked samples were tested in triplicate.

**Figure 7:**
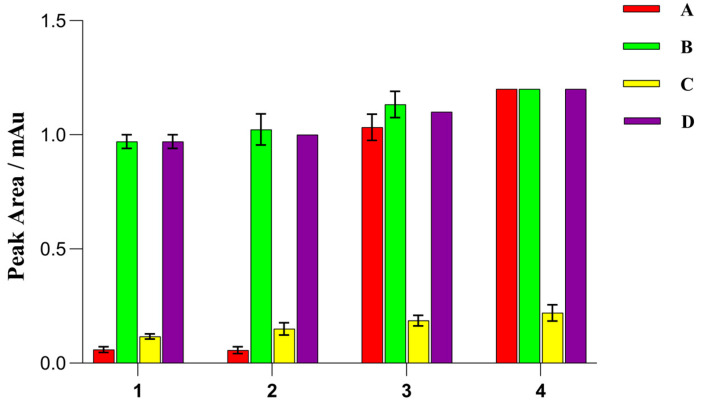
Effects of different extraction solvents and sorbents on the peak area of ochratoxin A (OTA). A, 30 mg of primary secondary amine (PSA) and 150 mg of MgSO_4_; B, 80 mg of graphite powder and 150 mg of MgSO_4_; C, 30 mg of graphitized carbon black (GCB) and 150 mg of MgSO_4_; D, 30 mg of octadecyl (C18) and 150 mg of MgSO_4_; 1, ACN; 2, ACN and 0.1% formic acid; 3, ACN and 1% formic acid; 4, ACN and 5% formic acid.

As shown in Figure 7, OTA was detected after extraction with all four solvents. ACN/formic acid (95/5, *v*/*v*) yielded the highest OTA peak area, but the obtained extracts exhibited a darker color compared to the other three extracts. Moreover, the use of ACN/formic acid (95/5, *v*/*v*) resulted in more interference from the coextracted materials during extraction. ACN/formic acid (99/1, *v*/*v*) yielded the second-highest OTA peak area. Thus, ACN/formic acid (99/1, *v*/*v*) was used as the extractant in subsequent studies.

#### 3.5.2. Selection of Cleaning Sorbent

It is imperative to perform the clean-up procedure during extraction to enhance the sensitivity, specificity, and purification degree [38,39]. Notably, the selection of the sorbent type is crucial, as it significantly impacts the clean-up procedure [38,39]. C18, PSA, and GCB are commonly used adsorbents for QuEChERS purification [40,41]. Therefore, the OTA clean-up effect of C18, PSA, and GCB was evaluated in this study. PSA, a weak anion exchange agent known for removing polar interferences, possesses excellent recovery and reproducibility when used for extracting various compounds with diverse properties [42]. C18 is employed for eliminating fats and non-polar compounds [43]. GCB exhibits a superior ability to remove pigments from grape samples compared to other sorbents, thereby yielding a more refined extract [12]. In this study, graphite powder was also used as a non-traditional purification agent. The chemical properties of graphite powder are relatively stable at room temperature. It is insoluble in water but soluble in acid, alkali, and organic solvents. Thus, 150 mg of anhydrous magnesium sulfate was combined with 30 mg of PSA, 30 mg of GCB, 30 mg of C18, and 80 mg of graphite powder, respectively, and used in the steps taken to purify the grape samples.

As shown in Figure 7, small OTA peak areas were obtained when GCB was used as the adsorbent. This can be attributed to the strong affinity between OTA and GCB, because the closer mycotoxin is to a planar structure, the stronger its affinity for GCB [44]. Conversely, large OTA peak areas were obtained when C18, PSA, and graphite powder were used as the adsorbents and when ACN/formic acid (99/1, *v*/*v*) was the extractant. However, C18 exhibited a limited ability to remove pigments, resulting in samples with a relatively dark color. Moreover, PSA is costly compared to graphite powder, thus making graphite powder a cost-effective alternative. Therefore, graphite powder was selected as the cleaning sorbent, and 150 mg of anhydrous magnesium sulfate and 80 mg of graphite powder were used in the purification procedure.

#### 3.5.3. Validation of the Method

The OTA levels in grapes were quantified using the optimized method. The calibration curves (working standard solution, grape matrices) covering a concentration range of 1–50 µg/kg for OTA exhibited excellent linearity and high correlation coefficients (R2= 0.9995, 0.9995). A low ME value of 7.11% was obtained for OTA [45]. The LOD and LOQ for OTA were 0.063 µg/kg and 0.21 μg/kg, respectively.

Table S3 shows the validation results for the precision and accuracy of the developed method. The OTA recoveries ranged from 82.61% to 119.69%, while the precision ranged from 1.53% to 7.48% for the three grape varieties across all spiking levels, indicating that the precision values were consistently below 10% [46]. Therefore, it can be inferred that the developed method has high accuracy and precision.

### 3.6. Determination of OTA Concentration

#### 3.6.1. QuEChERS Sample Preparation

Sample preparation was performed following the QuEChERS method. Briefly, grape berries were manually destemmed and deseeded and then homogenized to obtain the grape must. The must (10 g) was transferred to a 50 mL polypropylene tube. Then, 10 mL of acetonitrile with 1% formic acid was added, and the tube was vortexed for 5 min. Subsequently, 4.0 g of anhydrous magnesium sulfate and 1.0 g of sodium chloride were added, and the tube was vigorously shaken by hand for 3 min, followed by centrifugation at 8228× *g* for 5 min at 4 °C (model 5810R, Eppendorf, Hamburg, Germany). For the cleanup step, 1.0 mL of the supernatant was transferred to a 2.0 mL microtube containing 150 mg of anhydrous magnesium sulfate and 80 mg of graphite powder. Then, the mixture was vigorously shaken for 1 min, followed by centrifugation at 13,523× *g* for 30 s at 4 °C (model 5424R, Eppendorf, Hamburg, Germany). The obtained supernatant was filtered through a 0.22 μm PTFE membrane and then injected for HPLC-FLD analysis.

#### 3.6.2. OTA Quantification by HPLC-FLD

The OTA was quantified by HPLC-FLD using an Agilent 1260 Infinity II LC system (Agilent Technologies, Little Fall, DE, USA) and an Agilent Poroshell 120 EC-C18 column (3.0 mm × 150 mm, 2.7 μm) (Agilent Technology, Santa Clara, California, USA). The mobile phase consisted of 0.1% formic acid dissolved in water (A) and acetonitrile (B). The injection volume was 10 µL, and the flow rate was set at 0.5 mL/min. The column was maintained at 30 °C. The elution program was as follows: B: 0–5 min, 50%; 5–10 min, 70%; 10–15 min, 90%; 15–18 min, 50%. The excitation and emission wavelengths for fluorescence detection were 334 nm and 460 nm, respectively.

#### 3.6.3. Method Validation

The developed method was validated based on the evaluation of the following indices: limit of detection (LOD), limit of quantitation (LOQ), linear equation, linear range, correlation coefficient (r2), precision, accuracy, and matrix effect (ME). The standard curves were obtained by spiking different OTA concentrations in a solvent mixture (99:1 acetonitrile/formic acid). The OTA-free Merlot berries were considered as the blank matrix for the method validation.

For recovery studies, grapes of the Cabernet Gernischet, Cabernet Sauvignon, and red globe varieties were used as the representative matrices. Matrix-matched calibration curves were obtained using an OTA-free Merlot berries matrix. The linearity was determined using five matrix-matched calibration points for OTA. The LOD and LOQ were calculated using signal-to-noise ratios of 3 and 10, respectively. The precision and accuracy of the OTA method were assessed using relative standard deviation (RSD) and recovery [13].

The recovery was assessed at three spiking levels of quality control, with five replicates within the linear range of the calibration curve, i.e., 1.5 μg/kg, 15 μg/kg, and 40 μg/kg. The precision was determined from triplicate assays of grape berries spiked at three concentration levels (low, middle and high load of contamination), and was expressed as a percentage of RSD (%). The impact of the matrix effect (ME) was determined using the following equation: ME (%) = [(matrix-matched calibration curve slope—solvent curve slope)/solvent curve slope] [13,47].

### 3.7. Identification and Quantification of Volatile Compounds

#### 3.7.1. HS-SPME

The grape berries were manually destemmed and deseeded and then homogenized to obtain the must. The HS-SPME experimental parameters were adapted from a previous study with minor modifications [20,48]. Briefly, 5 g of grape berries, 2.5 g of sterile distilled water, and 1.5 g of NaCl were placed in a 20 mL headspace vial, and 10 µL of 4-methyl-2-pentanol solution (1.01 g/L) was added as the internal standard. Volatile compound extraction was performed using an AOC-6000 autosampler (Shimadzu, Basel, Switzerland) and an HS-SPME device coupled with a 50/30 µm DVB/CAR/PDMS fiber (Supelco Inc., Bellefonte, PA, USA) after equilibration at 45 °C for 10 min. Then, the fiber was inserted into the extraction head through a spacer, and the extraction was conducted for 30 min. Lastly, the fiber was immediately inserted into the GC injection port in splitless mode for 10 min to desorb the volatile compounds.

#### 3.7.2. DLLME

The grape berries were manually destemmed and deseeded, homogenized, and centrifuged at 4629× *g* for 10 min at 4 °C in a 5810 R centrifuge (Eppendorf AG, Hamburg, Germany). After discarding the supernatant, the DLLME procedure was performed according to the previously reported method with minor modifications [49]. Briefly, 2.5 mL of the supernatant, 2.5 mL of sterile distilled water, and 10 µL of 4-methyl-2-pentanol solution (1.02 g/L) were placed in a 10 mL centrifuge tube. Then, 1 mL of a solvent mixture containing an extractant (dichloromethane) and a dispersive solvent (acetone) at a ratio of 2:3 was added to each tube, followed by vigorous vortexing for 3 min. After the formation of a turbid suspension due to the dispersion of the dichloromethane droplets in the sample, the tubes were subjected to centrifugation at 4629× *g* for 10 min at 4 °C. The lower phase was collected, water was removed by adding anhydrous sodium sulfate, and the samples were filtered using a 0.22 µm PTFE membrane. Finally, 1.0 µL was injected into the injector port in 1:10 split mode for GC-MS analysis.

#### 3.7.3. GC-MS

GC-MS analysis was conducted using a GC-MS QP2010Ultra system (Shimadzu, Japan) and HP-INNOWax capillary column (60 m × 0.25 mm × 0.25 μm). High-purity helium (>99.99%) at a flow rate of 1.0 mL/min was used as the mobile phase. The temperature of the injector and transfer line was set at 250 °C. The GC program consisted of an initial temperature of 45 °C for 5 min, raised to 230 °C at a rate of 4 °C/min, and held for 5 min. The MS run program parameters consisted of the electron impact ionization mode, an ion source temperature of 200 °C, an m/z range of 30–500, and scanning at 0.3 s intervals. The retention indices (RI) were calculated after analyzing C7–C30 n-alkanes. The mass spectra were compared to those available in the National Institute of Standards and Technology (NIST) 20/20s database based on the RI of authenticated standards. The volatile compounds were quantified by comparing their peak areas with the peak area of the internal standard, and multiplying by the concentration of the internal standard.

### 3.8. Statistical Analysis

All measurements were performed in triplicate, and the results were presented as mean ± standard deviation. The volatile compounds identified in different grape samples were analyzed by *t*-tests using WPS software 16250 (Beijing Kingsoft Office Software, Inc., Beijing, China). The significance level was set at 5% (*p* < 0.05) for all statistical analyses. Principal component analysis (PCA) and orthogonal partial least squares discriminant analysis (OPLS-DA) were performed using SIMCA 14.1 software (Umetrics, Umea, Sweden). Figure 1 and Figure 2 were generated using Graph Pad Prism 9.4.0 software (GraphPad Software Inc., San Diego, CA, USA). The relationship between the volatile compounds and OTA was analyzed by Pearson correlation analysis (https://www.chiplot.online/ (accessed on 1 December 2023)) and the Pearson correlation coefficient was replaced by “r”.

## 4. Conclusions

In this study, the contents of OTA and volatile compounds in grapes contaminated with *Aspergillus carbonarius* were determined using a QuEChERS method coupled with HPLC-FLD. In the contaminated samples, OTA was initially detected on day 2 after contamination, indicating that day 1 was a crucial sampling point. Subsequently, the volatile compounds in grapes after day 1 of *A. carbonarius* inoculation were identified using HS-SPME-GC-MS and DLLME-GC-MS methods and analyzed using unsupervised PCA and supervised OPLS-DA. In particular, nine compounds, i.e., (*E*)-2-hexenal, styrene, trans-2-(2-pentenyl)furan, 1-hexanol, 3,7-dimethyl-3,6-octadienal, (*E*)-β-farnesene, nerolic acid, nerol, and ethyl acetate, were identified as significant differential volatiles in grapes contaminated with *A. carbonarius* before OTA biosynthesis. Finally, the correlation between significant differential volatiles and OTA was analyzed. The results showed correlations between ethyl acetate, styrene, 1-hexanol, (*E*)-2-hexenal, nerolic acid and OTA. This study provides evidence for the first time that ethyl acetate, styrene, 1-hexanol, (*E*)-2-hexenal and nerolic can be correlated with the OTA biosynthesis of *A. carbonarius* in ‘Muscat Hamburg’. In future studies, the DLLME procedure should be optimized to enhance the extraction efficiency of volatile compounds in contaminated grapes.

## Figures and Tables

**Table 1:** Significantly differential volatiles in grape samples CK1 and day 1 based on the analysis conducted using HS-SPME-GC-MS or DLLME-GC-MS data.

HS-SPME-GC-MS CK1d vs. 1d			DLLME-GC-MS CK1d vs. 1d		
(*E*)-2-hexenal	>	VIP = 1.25, *p* = 0.02	(*E*)-2-hexenal	>	VIP = 1.22, *p* = 0.04
styrene	>	VIP = 1.03, *p* = 0.01	nerol	>	VIP = 1.28, *p* < 0.01
trans-2-(2-pentenyl)furan	<	VIP = 1.25, *p* < 0.05	ethyl acetate	>	VIP = 1.25, *p* < 0.01
1-hexanol	<	VIP = 1.28, *p* < 0.05			
3,7-dimethyl-3,6-octadienal	<	VIP = 1.21, *p* = 0.04			
(*E*)-β-farnesene	>	VIP = 1.34, *p* < 0.05			
nerolic acid	>	VIP = 1.26, *p* < 0.05			

Volatiles were selected based on the VIP values (>1) of the OPLS-DA model and the *p*-values from *t*-tests (*p* < 0.05). The > symbol indicates that the volatile compound contents in the control group were significantly higher than those in the experimental group. The < symbol indicates that the volatile compound contents in the control group were significantly lower than those in the experimental group.

## Data Availability

Data are contained within the article and Supplementary Materials.

## Supplementary Materials

The following supporting information can be downloaded at: https://www.mdpi.com/article/10.3390/molecules29030567/s1, Table S1: Concentrations and RI of volatile compounds of samples from day 0, 1 and CK1 by HS-SPME-GC-MS; Table S2: Concentrations and RI of volatile compounds of samples from day 0, 1 and CK1 by DLLME-GC-MS; Table S3: Determination of OTA in grapes (*n* = 5); Figure S1: Supplementary figure for Figure 2; Figure S2: Supplementary figure for Figure 3.
