# Supplementary material for: Profiling of Volatile Compounds in ‘Muscat Hamburg’ Contaminated with Aspergillus carbonarius before OTA Biosynthesis Based on HS-SPME-GC-MS and DLLME-GC-MS
Source: Molecules. 2024 Jan 23;29(3):567. doi: 10.3390/molecules29030567 (PMC10856765; doi:10.3390/molecules29030567)
Supplement: Supplementary file 1 [file molecules-29-00567-s001.zip › molecules-2831858-supplementary.pdf]

Table S1. Concentrations and RI of volatile compounds of samples from day 0，1 and CK1 by HS-SPME-GC-MS.

NO	Compound	RI	Concentration (μg/kg)		
			0d	CK 1d	1d
1	hexanal	1077	1304.6±170.8	903.1±74.4	846.1±181.7
2	α-terpinene	1187	24.4±3.2	13.8±5.3	36.1±9.2
3	*D*-limonene	1206	207.7±16.4	167.0±16.8	318.4±110.1
4	β-phellandrene	1218	44.6±2.6	21.5±18.6	28.6±15.3
5	(*E*)-2-hexenal	1228	882.6±117.3	633.2±55.1	406.7±91.0
6	trans-β-ocimene	1242	149.7±12.2	185.6±4.5	166.2±107.8
7	cis-β-ocimene	1259	270.2±14.5	219.3±10.7	396.2±112.4
8	styrene	1269	34.1±1.6	46.7±5.6	38.1±7.1
9	*p*-cymene	1280	3.3±2.8	3.1±2.7	4.1±1.9
10	terpinolene	1287	61.8±6.2	44.2±0.3	90.5±27.8
11	trans-2-(2-pentenyl)furan	1311	13.8±2.4	14.2±3.5	27.8±5.4
12	6-methyl-5-hepten-2-one	1345	34.6±3.8	26.0±0.7	24.3±1.3
13	1-hexanol	1355	68.5±6.5	58.1±5.3	72.0±2.4
14	trans-rose oxide	1359	65.6±4.3	60.3±5.6	58.0±13.8
15	cis-rose oxide	1377	18.0±1.1	19.1±1.8	20.4±6.6
16	allo-ocimene	1379	70.0±4.4	64.2±6.7	106.0±33.2
17	(*Z*)-3-hexen-1-ol	1388	4.1±0.4	3.8±0.6	6.2±4.6
18	nonanal	1402	45.4±4.6	36.5±3.0	24.6±7.9
19	(*Z*)-2-hexen-1-ol	1409	32.8±1.3	37.9±1.3	41.1±12.4
20	(*E,E*)-2,4-hexadienal	1416	9.2±1.4	6.8±0.6	8.4±6.2
21	4-ethyl-2-hexynal	1426	13.1±0.9	17.0±1.0	23.9±11.5
22	perillen	1430	6.1±0.6	12.5±0.9	16.1±8.2
23	(*E*)-2-octenal	1441	12.6±0.5	24.2±0.8	17.8±8.2
24	linalool oxide (furanoid)	1450	65.9±3.6	56.1±2.4	87.9±30.8
25	1-octen-3-ol	1453	7.3±0.2	15.2±6.0	11.7±2.6
26	6-methyl-5-hepten-2-ol	1465	31.8±0.8	45.0±2.7	55.8±6.0
27	citronellal	1489	2.1±0.2	5.4±1.0	2.4±0.5
28	2-ethyl-1-hexanol	1492	5.3±0.3	6.7±0.8	7.0±2.0
29	(*E,E*)-2,4-heptadienal	1507	9.4±0.7	11.5±0.9	10.0±2.9
30	benzaldehyde	1543	5.8±0.5	9.1±0.4	6.9±1.2
31	(*E*)-2-nonenal	1547	12.1±2.4	19.0±5.2	12.2±2.6
32	linalool	1553	6719.5±101.7	5573.3±409.8	8732.2±2735.7
33	3,7-dimethyl-3,6-octadienal	1572	1.6±0.2	0.7±0.6	3.0±1.3
34	(*E,Z*)-2,6-nonadienal	1600	10.9±2.5	9.6±2.8	6.5±0.3
35	hotrienol	1615	21.4±4.8	13.7±0.8	16.6±6.4
36	(*E*)-2-octen-1-ol	1620	1.3±1.1	2.1±1.2	0.8±0.7
37	decanoic acid ethyl ester	1644	7.2±2.7	7.9±3.5	12.3±6.4
38	benzeneacetaldehyde	1665	31.3±1.8	33.2±4.4	38.0±4.3
39	citronellyl acetate	1669	3.1±0.3	3.3±0.6	2.1±1.9

40	(*E*)-β-farnesene	1673	32.2±9.5	29.0±0.8	16.5±4.6
41	neral	1696	20.0±0.9	26.9±2.5	38.5±16.7
42	α-terpineol	1706	174.6±19.4	105.8±3.6	84.4±25.8
43	α-cyclogeraniol	1714	6.6±1.3	6.3±1.2	8.1±0.7
44	trans-citral	1746	39.9±1.5	49.4±1.5	61.9±12.3
45	trans-pyranoid linalool oxide	1748	41.3±15.8	37.3±1.1	17.6±15.3
46	α-citronellol	1762	4.6±0.8	4.4±0.5	4.1±1.8
47	geranyl acetate	1765	1.4±0.2	2.1±0.2	3.0±1.9
48	β-citronellol	1770	413.9±4.9	324.0±7.9	248.9±67.2
49	7-methyl-3-methylene-6-octen-1-ol	1793	28.9±0.2	23.5±1.1	18.0±3.9
50	nerol	1807	543.4±15.8	501.5±31.5	505.2±84.5
51	isogeraniol	1818	7.4±0.2	7.1±1.2	6.3±1.8
52	trans-geraniol	1853	1076.2±23.0	1026.7±63.1	944.3±99.0
53	isopulegol	1861	14.1±4.5	12.4±6.7	9.1±3.3
54	benzyl alcohol	1881	4.8±1.0	4.9±0.6	4.7±0.6
55	phenylethyl alcohol	1931	4.9±0.6	5.1±0.5	7.2±1.8
56	nerolic acid	2349	18.0±0.7	50.8±15.4	14.4±2.3

a Values shown represent the averages of triplicate samples, results were expressed as the mean value ± standard deviation (SD).

b Volatile compounds of grape samples from day 0, 1 and CK1 : namely 0d, 1d, CK1d.

Table S2. Concentrations and RI of volatile compounds of samples from day 0, 1 and CK1 by DLLME-GC-MS.

NO	Compound	RI	Concentration (μg/kg)		
			0 d	CK 1d	1 d
57	ethyl acetate	903	216.2±18.8	222.2±38.2	91.7±17.2
58	butyl acetate	1066	411.7±22.4	456.5±40.0	440.1±28.9
59	hexanal	1077	254.1±30.7	131.7±55.0	292.9±46.6
60	(*E*)-2-hexenal	1228	207.6±25.1	186.0±21.6	67.6±42.1
61	linalool	1553	1037.6±27.4	1429.0±133.9	2716.8±549.2
62	trans-pyranoid linalool oxide	1748	277.9±70.7	184.4±26.8	230.6±55.6
63	β-citronellol	1770	196.2±24.9	278.0±22.8	205.7±41.3
64	nerol	1807	632.3±73.8	1750.0±141.6	947.6±137.4
65	trans-geraniol	1853	2089.5±146.9	19995.9±110.5	2258.4±157.2
66	benzyl alcohol	1881	51.6±10.2	127.5±32.4	86.5±27.5
67	phenylethyl alcohol	1931	93.0±31.2	174.4±40.1	121.0±0.6
68	(*E*)-2,6-dimethyl-3,7-octadiene-2,6-diol	1938	121.3±54.4	106.3±28.8	198.3±55.5
69	2,6-dimethyl-1,7-octadiene-3,6-diol	2104	87.5±19.3	139.2±89.0	135.2±38.2
70	2,4-di-tert-butylphenol	2326	7762.8±860.3	12168.8±4481.6	9891.0±1692.9
71	nerolic acid	2349	1518.4±56.2	2864.3±112.4	2697.0±409.5

a Values shown represent the averages of triplicate samples, results were expressed as the mean value ± standard deviation (SD).

b Volatile compounds of grape samples from day 0, 1 and CK1: namely 0d, 1d, CK1d.

Table S3. Determination of OTA in grapes (n=5)

	Spiked level (μg/kg)	Grape		
		Cabernet Gernischt	Cabernet Sauvignon	Red Globe
Recovery (%)	1.5	82.61	106.88	107.92
	15	116.49	118.93	119.69
	40	105.94	109.48	110.58
RSD (%)	1.5	4.71	4.32	7.48
	15	4.35	3.07	3.96
	40	3.64	1.53	2.00

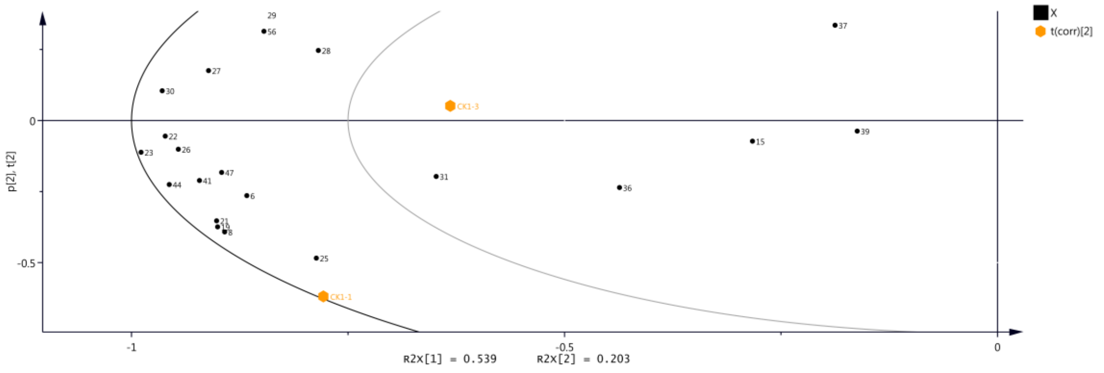

Figure S1. Supplementary figure for Figure 2.

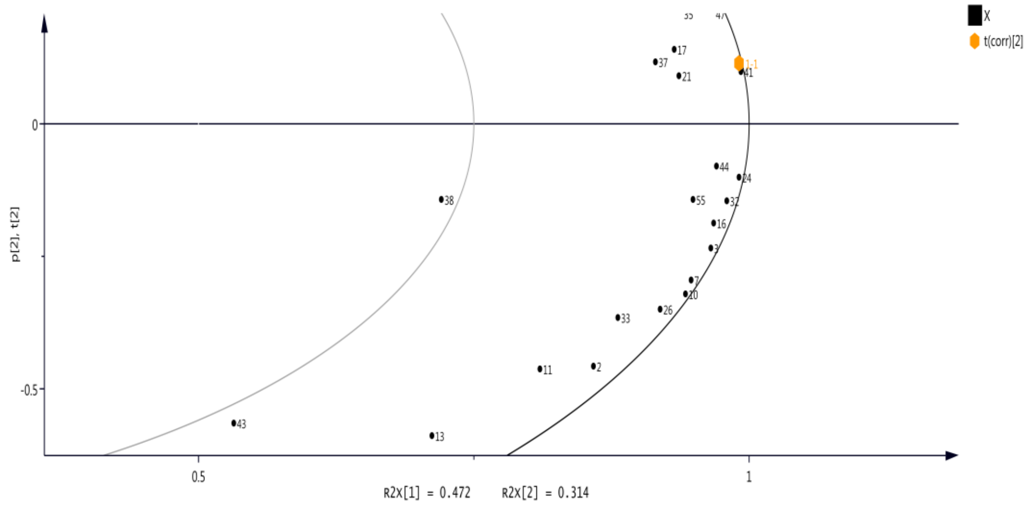

Figure S2. Supplementary figure for Figure 3.
